# Bacterial resistance to temperate phage is influenced by the frequency of lysogenic establishment

**DOI:** 10.1016/j.isci.2024.109595

**Published:** 2024-03-27

**Authors:** Hiba Baaziz, Rita Makhlouf, Michael McClelland, Bryan B. Hsu

**Affiliations:** 1 Department of Biological Sciences, Fralin Life Sciences Institute, Center for Emerging, and Zoonotic, Arthropod-borne Pathogens, Virginia Tech, Blacksburg, VA 24061, USA; 2 Department of Microbiology and Molecular Genetics, School of Medicine, University of California, Irvine, Irvine, CA, USA

**Keywords:** Virology, Microbiology, Viral microbiology

## Abstract

Temperate phages can shape bacterial community dynamics and evolution through lytic and lysogenic life cycles. In response, bacteria that resist phage infection can emerge. This study explores phage-based factors that influence bacterial resistance using a model system of temperate P22 phage and *Salmonella* both inside and outside the mammalian host. Phages that remained functional despite gene deletions had minimal impact on lysogeny and phage resistance except for deletions in the *immI* region that substantially reduced lysogeny and increased phage resistance to levels comparable to that observed with an obligately lytic P22. This *immI* deletion does not make the lysogen less competitive but instead increases the frequency of bacterial lysis. Thus, subtle changes in the balance between lysis and lysogeny during the initial stages of infection can significantly influence the extent of phage resistance in the bacterial population. Our work highlights the complex nature of the phage-bacteria-mammalian host triad.

## Highlights

• Deletion of the P22 immunity region, *ImmI*, decreases its frequency of lysogeny in STm
• Increased lysis by P22 mutants leads to greater phage resistance among STm
• Increased phage resistance among STm is observed in culture and mice

## Graphical abstract

**Figure:**
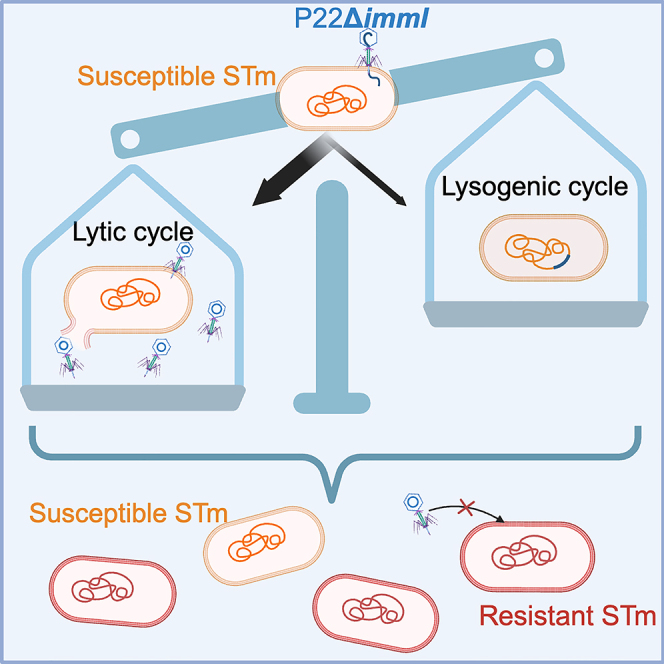

## Introduction

Temperate phages are bacterial viruses that can initiate either lytic or lysogenic cycles after infection. In addition to lysing the cell when producing progeny (“lytic”), temperate phages can integrate their genome into the bacterial chromosome as a prophage (“lysogenic”). This can provide bacterial hosts with ecological and evolutionary benefits that include expanding their metabolic repertoire and enhancing virulence, which ultimately improves competitive fitness.^1^ Moreover, prophages can protect their bacterial hosts from other phages by disrupting superinfection via homoimmunity, exclusion, and restriction mechanisms.^2^ Reflective of their significance, prophages are highly prevalent in the mammalian gut^3^ and are major contributors to the pool of free phage particles, through spontaneous induction.^4^

In the early stages of infection, temperate phages must make a pivotal decision between lysis and lysogeny that is typically governed by various factors, which include nutrient availability, multiplicity of infection,^5^^,^^6^ and signaling molecules.^7^^,^^8^ Interestingly, at a population level, this decision-making process is not strictly in one direction. Genetically identical cells that are infected by the same phage can opt for different outcomes, which leads to a mixture of lysogenized and lysed cells.^5^^,^^9^ At a molecular level, this decision is often modulated by phage-encoded regulatory proteins, of which the most well known is the cI-cro genetic switch of lambdoid phages.^10^ For the *Salmonella* lambdoid phage, P22, lysogeny is maintained by the C2 protein repressor, which blocks expression of the early genes that initiate lysis. A transition toward lysis is initiated by the inhibition of the C2 protein function, which de-represses expression of the Cro protein.^11^^,^^12^

Although the mechanisms of lysis and lysogeny have been studied for decades, the factors that influence the establishment of lysogeny soon after infection are less understood. With infection by virulent phages, which obligately leads to cell lysis, bacteria often develop resistance through various mechanisms such as the inhibition of adsorption, interference with DNA injection, degradation of injected DNA, or abortive infection.^13^ In such cases, there are clear benefits for the bacterial host to prevent virulent phage infection and subsequent cell lysis. However, with temperate phages, where the outcome of infection could benefit the host via lysogeny, the extent to which bacteria develop resistance is less clear.

Herein, we investigate the phage-based factors that influence bacterial resistance to temperate phage. Using the model temperate phage, P22, we generated deletions in regions known to be non-essential to phage function and investigated how these deletions influence the frequency of lysogeny and phage resistance in the host bacterium, *Salmonella enterica* serovar Typhimurium (STm). We found that most deletions did not significantly alter the frequencies of lysogeny or phage resistance compared with wild-type P22, *in vitro* and *in vivo*. Interestingly, P22 mutants lacking the *immI* region, which expresses proteins that modulate C2-Cro regulation of lysogeny, resulted in low lysogeny and high phage resistance in the bacterial host. Further investigation showed that the presence of the P22-*immI*-deleted region in lysogens neither hindered bacterial growth nor impeded phage production. When grown in competition with P22-resistant bacteria, these lysogens did not exhibit impaired fitness. Finally, we measured the frequency of lysogeny established upon infection and found that the absence of the *immI* region reduces the frequency of lysogeny, leading to a greater frequency of bacterial lysis, thus enriching for phage-resistant strains. Collectively our work describes how lysis and lysogeny are carefully balanced at the initial stages of infection, and modulation of this balance toward increased lysis can not only reduce the frequency of lysogeny but also lead to increased phage resistance that rivals the effect of an obligately lytic P22 phage.

## Results

### Viable phage particles can be produced from deletion mutants of P22 prophages

**Figure 1:**
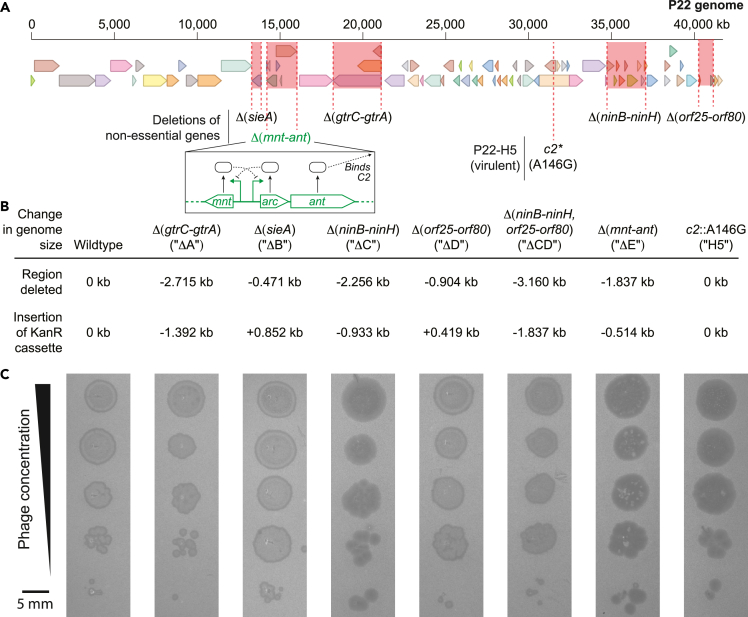
Construction of P22 mutants (A) Map of the P22 genome with deletions represented in red highlighted regions. (B) Change in genome size after region replacement with KmR cassette. (C) Representative pictures of plaque morphology of P22 deletion mutants against STm.

We generated deletion mutants in regions of P22 phage that were previously reported as non-essential to phage function, replacing them with a kanamycin resistance cassette. The specific regions deleted are shown in Figure 1A: P22ΔA refers to *gtrC-gtrA*, which encodes proteins that alter the bacterial *O*-antigen to inhibit adsorption by superinfecting phage^14^; P22ΔB refers to *sieA*, which encodes an inner membrane protein that acts to exclude superinfecting phage^15^; P22ΔC refers to *ninB-ninH*, which encodes proteins involved in *N*-independent transcriptional termination^16^; P22ΔD refers to *orf25-orf80* whose products facilitate a pseudo-lysogenic state after phage infection^17^; and P22ΔE refers to *mnt-ant*, which acts as a secondary immunity region (“*immI*”). Proteins encoded by the *immI* region are known to modulate the lysis-lysogeny decision-making of the primary immunity region in P22 phage (“*immC*”).^18^ We generated deletions in two additional regions: *16-sieA-mnt-ant-9* and *eaC-eaE*, but neither prophages were able to generate viable phage particles. All deleted regions in P22 prophages were replaced with kanamycin resistance (KmR) cassettes using lambda-red recombination.^19^ Most deletion/insertion modifications reduced P22 genome sizes, and the net increases in P22ΔB and P22ΔD phages (Figure 1B) remained below the ∼1.6 kb terminal redundancy of the P22,^20^ thus ensuring the successful encapsidation of the complete genome. We also characterized the lytic mutant of P22 phage, P22-H5, which contains a non-synonymous mutation (AAC -> AGC) in the phage repressor gene, *c2*. Notably, all mutant P22 phages we generated are capable of lysogenization and lysis of STm (Figure 1C).

### Lysogeny and phage resistance of STm varies between P22 mutants

**Figure 2:**
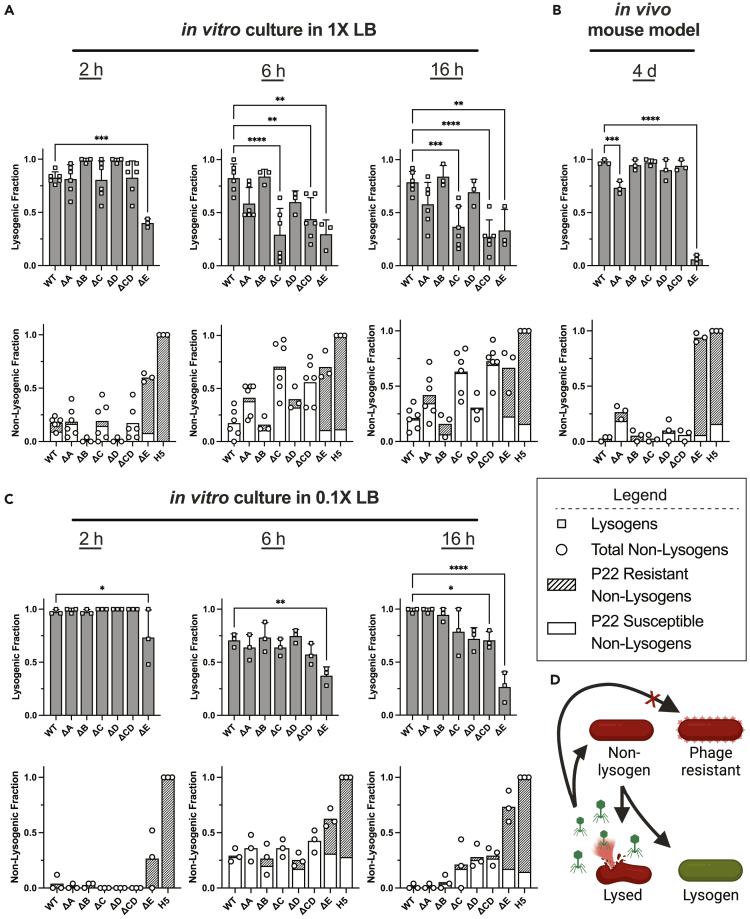
Lysogeny of mutant P22 phages *in vitro* and *in vivo* (A) Mixtures of STm with phages *in vitro* in 1X LB, (B) *in vivo*, and (C) *in vitro* in 0.1X LB were analyzed for the proportion of STm colonize that were lysogenized by phage, non-lysogenic and resistant to phage, or non-lysogenic and susceptible to phage. (D) Upon infection of non-lysogenic bacteria, temperate phages follow either a lytic cycle in which they replicate within bacterial cells and release phage progeny or a lysogenic cycle in which they integrate the bacterial genome and replicate with the bacterial host. Additionally, bacteria may be resistant to phage infection, leading to inhibition of phage propagation and cell lysis. Bars represent means and error bars represent standard deviations. Asterisks denote p-values determined by one-way ANOVA with Dunnett’s multiple comparisons to control (wild-type P22) test. ∗p < 0.05; ∗∗p < 0.005; ∗∗∗p < 0.0005; ∗∗∗∗p < 0.0001.

The impact of P22 phage mutants on a bacterial host was investigated by coculturing them separately with STm to quantify lysogeny and emergence of phage resistance. Wild-type P22 and P22-H5 were used as controls. As shown in Figure 2A, lysogeny was generally high in the first 2 h but dropped by 6 h and 16 h. Among P22 deletion mutants, P22ΔC, P22ΔCD, and P22ΔE phages exhibited significantly lower levels of lysogeny compared with wild-type P22 phage. This reduction in lysogeny by P22ΔC and P22ΔE phages was also evident in plaque morphologies shown in Figure 1C, where the reduced turbidity led to plaques resembling those produced by the lytic P22-H5 phage.

Both P22ΔC and P22ΔCD mutants lack *ninBDEXFGYH*, which contains terminators that end transcription prior to reaching gene *23*. Studies of the analogous *nin*-region in λ phage have shown that about half of early right-ward transcription originating from pR terminates prior to gene *Q*^21^ (analogous to gene *23* in P22 phage) and the deletion of the λ *nin*-region leads to readthrough into gene Q.^16^^,^^22^ This premature expression of gene *Q* negatively affects lysogeny by λ phage,^23^ and so we suspect a similar readthrough to gene *23* in P22ΔC and P22ΔCD phages leads to the reduced lysogeny we observed. The P22 *immI* region encodes an anti-repressor protein, Ant, that binds to the C2 protein and can inhibit its repressor activity when overexpressed,^24^ protects it from RecA-mediated proteolysis,^25^ and acts to inhibit superinfecting phage.^26^ Studies revealed that this region acts on the *immC* region but is dispensable for lysis and lysogeny.^18^ Although we find this to be qualitatively true, quantitation of the bacterial impact from P22ΔE phage (i.e., lacking the *immI* region) revealed significant reductions in lysogeny and increases in resistance to P22ΔE phage, reaching levels comparable to P22-H5 phage. Cultures of P22 mutants with STm showed generally comparable microbial concentrations with exceptions for P22ΔE and P22-H5 phages, which had lower bacterial concentrations and higher phage concentrations (Figure S1).

Because the lysis-lysogeny decision and phage resistance evolution *in vitro* are not necessarily reflective of *in vivo* outcomes,^27^^,^^28^^,^^29^ we determined whether the *in vitro* effects of P22 mutants were relevant *in vivo*. We quantified lysogeny and phage resistance in STm by using a murine model of *Salmonella* infection.^30^ Previously, we found that fecal concentrations of STm and wild-type P22 stabilize by day 4 post-infection (Figure S2). As shown in Figure 2B, lysogeny was high for nearly all P22 mutants except for P22ΔE phage, which exhibited a substantial reduction in lysogeny. Similar to *in vitro* experiments with P22ΔE phage, there was a high frequency of P22 resistance among non-lysogens of STm on par with levels observed with P22-H5.

When comparing the fraction of lysogens resulting from different P22 mutants *in vivo* to the results *in vitro*, it became evident that the latter has markedly lower lysogeny. Lysogeny by lambdoid phages is affected by several factors including nutrients, multiplicity of infection, and bacterial cell size.^31^^,^^32^ In the mammalian gut, the acquisition of nutrients is highly contested.^33^^,^^34^ Consequently, we investigated whether a less nutritious medium, 10% LB (0.1X LB), would lead to higher degrees of lysogeny *in vitro*, similar to our *in vivo* observations. As shown in Figure 2C, lysogeny was generally higher for all time points in lower nutrient. Notably, the substantially lower lysogenic fraction by P22ΔE phage and high levels of P22 resistance in non-lysogenic STm cultured with P22ΔE and P22-H5 phages were observed again.

### Resistance to P22 phage does not reduce the competitive fitness of STm *in vitro*

**Figure 3:**
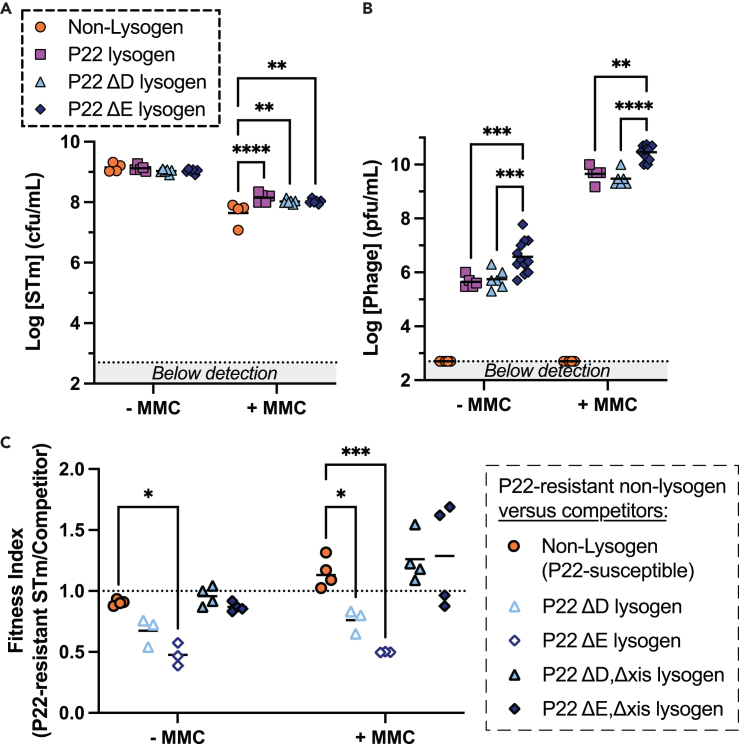
Mutations in lysogens do not alter bacterial growth but can lead to increased phage production (A) Bacterial and (B) phage concentrations after overnight monoculture of STm non-lysogens and lysogens. (C) Coculture competition of individual STm non-lysogens and lysogens against P22-resistant *rfbP mutant* STm. Lines represent means. Asterisks denote p-values determined by two-way ANOVA with Tukey’s multiple comparisons test. ∗p < 0.05; ∗∗p < 0.005; ∗∗∗p < 0.0005; ∗∗∗∗p < 0.0001.

The consistently high frequency of phage resistance in STm against P22ΔE phage, despite the phage’s ability to lysogenize STm, was unique to the P22ΔE mutant and reproducible *in vivo* and across different culture conditions *in vitro*. To understand the factors involved in the development of resistance to a temperate phage that remains capable of establishing lysogeny, we hypothesized that a phage-resistant STm would have greater fitness than the other bacteria present: non-lysogenic STm and STm lysogenized by P22ΔE (Figure 2D). To test this hypothesis, we investigated whether the P22ΔE prophage conferred disadvantages to STm by assessing the growth and phage production of a P22ΔE lysogen in various conditions. Wild-type P22 and P22ΔD lysogens were used as controls. As shown in Figure 3A, we found that the growth of overnight cultures of all lysogens were comparable. Growth curves of each lysogen were similarly comparable (Figure S3). Even under inducing conditions with mitomycin C (MMC), lysogens had higher bacterial concentrations compared with the non-lysogenic STm. Quantification of the phage titers (Figure 3B) showed that P22ΔE lysogens produced higher phage concentrations than wild-type P22 and P22ΔD lysogens. Overall, these results indicate that deletion of *immI* in P22ΔE lysogens is not detrimental to bacterial growth or phage production in monoculture.

In the absence of obvious detriments to bacterial growth or phage production, we investigated whether direct competition with a P22-resistant STm (Δ*rfbP*) would impair the growth of P22ΔE lysogen under different conditions. Non-lysogenic STm and P22ΔD lysogen were used as controls. For these coculture competition experiments, we calculated the Fitness Index of non-lysogenic or lysogenic STm against P22-resistant non-lysogenic STm. Because of the susceptibility of non-lysogenic STm to P22, we could not use this strain as a reference in competition against lysogens and thus used P22-resistant STm. As shown in Figure 3C, the P22-resistant strain had comparable fitness to a non-lysogenic STm but was less competitive against P22ΔD and P22ΔE lysogens (Fitness Index <1), even under inducing conditions. To determine if phage produced from these lysogens could impede growth of the P22-resistant STm, we deleted the excisionase gene, *xis*, to prevent the excision of P22ΔD and P22ΔE prophages and inhibit phage production.^35^ Competition of P22-resistant STm against P22Δ (D,*xis*) and P22Δ (E,*xis*) lysogens showed that although the lysogens lost their fitness advantages, the P22-resistant STm still does not have a superior Fitness Index. Interestingly, during MMC induction, the *xis*^*—*^ lysogens were generally less competitive. This could be attributed to escape replication where prophage replication continues into the bacterial genome to produce an unusable product,^35^^,^^36^ siphoning cellular resources away from bacterial growth.^9^ Nonetheless, it remains notable that the deletion of the *immI* is not detrimental to fitness of its lysogen and does not explain the enrichment of phage resistance in STm.

### P22ΔE phage establishes lysogeny of STm at a low frequency

**Figure 4:**
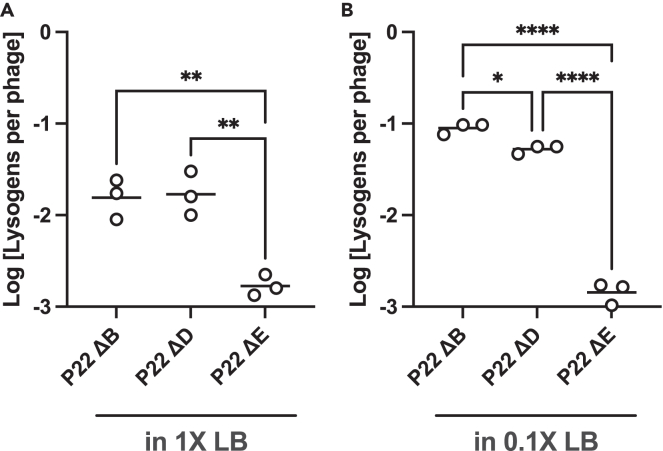
P22ΔE phage establishes lysogeny of STm at a low frequency Deleting the *immI* region leads to a reduced frequency of lysogeny compared with other P22 phages in both conditions: (A) 1X LB and (B) 0.1X LB. Lines represent means. Asterisks denote p-values determined by one-way ANOVA with Tukey’s multiple comparisons test. ∗p < 0.05; ∗∗p < 0.005; ∗∗∗p < 0.0005; ∗∗∗∗p < 0.0001.

After finding that phage-resistant strains of STm do not exhibit greater competitiveness against P22ΔE lysogens, our subsequent hypothesis was that the deletion of *immI* alters the frequency at which lysogeny is established. We investigated this hypothesis via one-step infection of wild-type STm by P22ΔE phage. P22ΔB and P22ΔD mutants were used as controls because they both had high levels of lysogeny and low levels of resistance development in STm (Figure 2). As shown in Figure 4A, we found that, out of the total number of active phage infections (lytic or lysogenic) determined by plaque assay, the control mutants P22ΔB and P22ΔD phages lysogenized STm at 1.7 ± 0.8% and 1.9 ± 1.0%, respectively, whereas P22ΔE phage had a significantly lower number of lysogens per phage at 0.17 ± 0.05% (mean ± SD). With diluted nutrient conditions of 0.1X LB (Figure 4B), which we found to promote lysogeny *in vitro* (Figure 2C), P22ΔB and P22ΔD phages had increased lysogeny per phage (9.0 ± 1.2% and 5.3 ± 0.5%, respectively), whereas lysogeny by P22ΔE phage remained unaffected (0.15 ± 0.04%), indicating that lysogeny established by P22ΔE at a low frequency is independent of nutrient availability. With reduced lysogeny, the alternative pathway for temperate phages is lysis, and thus these results indicate that this shift away from lysogeny toward lysis may be a factor in the enrichment of phage-resistant bacteria.

### P22ΔE phage promotes the enrichment of phage-resistant bacteria

**Figure 5:**
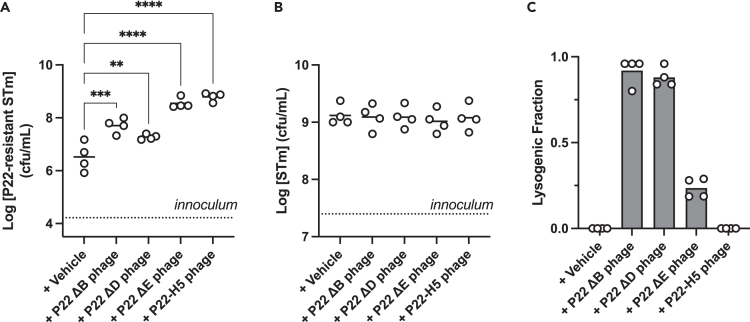
Impact of phage on fitness of resistant strains (A) Deleting the *immI* region leads to an increase in P22-resistant STm. (B) Addition of P22 phage to cocultures of various STm strains versus P22-resistant STm can be advantageous for either lysogeny or P22 resistance. (C) Deleting the *immI* region leads to a reduced frequency of lysogeny compared with other P22 phages. Lines and bars represent means. Asterisks denote p-values determined by one-way ANOVA with Dunnett’s multiple comparisons to control (“vehicle”) test. ∗p < 0.05; ∗∗p < 0.005; ∗∗∗p < 0.0005; ∗∗∗∗p < 0.0001.

To understand the association between low lysogeny frequency and high resistance, we hypothesized that the reduced lysogeny and increased lysis by P22ΔE phage would lead to the expansion of phage-resistant STm. To test this, we added P22ΔE phage to a coculture of wild-type STm and P22-resistant STm inoculated at a 1000:1 ratio. P22ΔB and P22ΔD were used as controls. As shown in Figure 5A, the addition of P22ΔB and P22ΔD phages led to ∼15-fold and ∼5-fold increases in P22-resistant STm compared with the buffer vehicle, respectively. By contrast, P22ΔE led to a ∼100-fold increase, which was comparable to the effect of the phage repressor gene mutant P22-H5 that resulted in ∼190-fold increase. The concentration of wild-type STm was unchanged between conditions (Figure 5B). We assessed the lysogenization of STm wild-type by phage mutants. Results showed high lysogeny by P22ΔB and P22ΔD phages and low lysogeny by P22ΔE phage (Figure 5C), which was consistent with our previous observations (Figure 2). Collectively, the low frequency of lysogeny and increased lysis during one-step infection by P22ΔE phage and the resultant enrichment of phage resistance in STm reveals that the frequency of lytic replication by temperate phage P22 influences the compositional makeup of the bacterial host (e.g., non-lysogen, lysogen, phage-resistant), *in vitro* and *in vivo*.

## Discussion

Our study sheds light on the complex interplay between lysis and lysogeny for temperate phages, revealing how this decision-making at the onset of infection can result in altered frequencies of phage resistance among targeted bacteria. Among deletion mutants in P22, we found that P22ΔE phage, which lacks the secondary immunity region *immI*, had reduced lysogeny and increased phage resistance in STm *in vitro* and within the mammalian gut. Investigating the link between reduced lysogeny and increased resistance revealed that reduced frequency in lysogenic establishment and increased lysis resulted in the enrichment of phage resistance among STm, to levels similarly observed with the lytic P22-H5 phage. To our knowledge, this is the first quantitative description of how consequential the lytic/lysogenic balance is for temperate phages and lysogeny: a shift toward lysis increases the frequency of phage resistance and reduces the frequency of lysogeny among bacteria.

Research into phage resistance among bacteria has led to several important discoveries, many of which relate to virulent phages. This focus stems from the great challenge that resistance poses to the sustainable development and success of phage therapy, an approach that relies on virulent phages to eradicate the targeted bacteria.^37^ In contrast, the extent to which bacteria develop resistance to phages that are not obligately lytic, i.e., temperate phages, is less robust. Lysogeny can benefit the bacterial host by conferring fitness advantages within competitive environments like the mammalian gut. In some cases, this lysogeny by temperate phages can take precedence over CRISPR-Cas bacterial immunity that otherwise inhibits lytic mutants.^38^ Additionally, it has been shown that horizontal gene transfer mediated by temperate phages can provide a directional selection that improves bacterial adaptation and fitness in the mammalian gut.^39^

In the present work, we show that reducing the frequency of lysogenic establishment by temperate phages can have downstream effects that ultimately leads to an increased frequency of phage resistance. Although there are several resistance mechanisms, mutation of the phage receptor in bacteria is a primary route.^40^ Resistance to lytic phage often reduces bacterial host fitness,^41^ such as in the case with *Escherichia coli* strains resistant to T-type lytic phages.^42^ Interestingly, the high resistance observed in non-lysogenic STm cells was not due to decreased fitness of P22ΔE lysogens. In fact, the P22ΔE lysogen displayed a fitness advantage over the phage-resistant strain, reaching levels comparable to that of a non-lysogenic STm. In STm, the O-antigen of lipopolysaccharide (LPS) serves as a receptor for P22 phage.^11^^,^^43^ Transposon library screens have shown that certain non-essential genes associated with O-antigen and LPS biosynthesis can be deleted to provide resistance to P22 infection.^44^^,^^45^ Recently, Berryhill et al. used model-based and *in vitro* experiments to show that temperate λ phage leads to similarly high levels of lysogenic and phage-resistant non-lysogenic *E. coli* after one day, whereas the presence of virulent λ phage results in a numerical dominance of the latter.^46^ Not only are our results consistent with these observations, but we also show that even an increased frequency of lysis by temperate phages can lead to elevated phage resistance, reaching levels similar to those seen with a strictly lytic phage. For a greater understanding of this relationship between lysis/lysogeny by temperate phages and phage resistance in the host bacterium, more detailed genetic studies will be needed.

Although the molecular mechanisms of lysis and lysogeny are well studied for lambdoid phages,^47^ the factors that influence bacterial resistance to such phages are not well understood. The *immI* region is not essential for lysogeny, but our data indicate it is important for modulating the frequency of its establishment. Encoded in this region are *mnt*, *arc*, and *sar*, which act to regulate the expression of *ant*, a protein antirepressor (Figure 1A). *Mnt* encodes a protein that transcriptionally represses expression of *arc* and *ant*,^48^ *sar* encodes antisense RNA that binds to *ant* mRNA,^49^ and *arc* encodes a repressor that inhibits *mnt* expression^48^ and moderates *ant* expression.^26^ The Ant protein complexes C2, which protects it from autoproteolysis induced by activated RecA under DNA-damaging conditions.^25^^,^^50^^,^^51^ Although lysogeny can be maintained by C2 in the presence of endogenous levels of Ant,^52^ the overexpression of Ant can inhibit C2 binding to O_R_ and O_L_ operators and de-repress *cro* expression to trigger the lytic cycle.^24^ Past study has shown that C2 levels are elevated during the initial stages of infection followed by a lowering during lysogenic maintenance,^53^ which suggests the importance of concentration. Presently, it is unclear how the deletion of the *immI* region in P22ΔE phage reduces the frequency of lysogeny in STm but we hypothesize that Ant may have a dynamic role that is influential during the establishment of lysogeny.

Overall, this work highlights how changes in the delicate balance between lysis and lysogeny at the onset of infection can shape the final composition of the bacterial host community. Although our study describes a population-wide impact of a seemingly subtle shift in the initial decision-making of phages during infection, it reveals several new questions. For example, what are the long-term evolutionary consequences of bacterial resistance to temperate phages, and how does it impact the coevolutionary dynamics between bacteria and phages? Is there an ecosystem-specific “ideal” frequency of lysogeny that optimizes for the production and dissemination of free phages, and is this common among temperate phages within the same ecosystem? Understanding these mechanisms not only enhances our knowledge of microbial interactions but also holds significant biomedical relevance. Several temperate phages are known to encode clinically relevant virulence factors such as toxins (e.g., Shiga, cholera, diphtheria, and botulism)^54^ that are produced during the lytic life cycle but not as prophages. Additionally, temperate phages can be used to genetically modify bacteria within *in vivo* environments.^55^ Collectively, greater comprehension of the dynamics of phage-host interactions has both ecological and biomedical importance.

### Limitations of the study

To decipher the underlying mechanisms that contribute to the observed reduction in lysogenic frequency by P22ΔE phage, it will be important to further assess the impact of various mutations within the two immunity regions, *immI* and *immC*, on the lysis-lysogeny balance in STm. One limitation to our study is that we do not investigate the effects that deleting *immI* has on *immC*, specifically C2 and Cro expression levels. From the study of another lambdoid phage, lambda, a cell contains a small number (∼150) of the CI phage repressor protein^56^ (equivalent to C2 in P22 phage). Additionally, cell fate after phage infection is highly sensitive to CI concentrations, i.e., smaller cells tend toward lysogeny, whereas larger cells tend toward lysis.^31^ Thus, even subtle alterations in phage repressor (CI or C2) concentrations, whether through the mutagenesis of endogenous operators or the introduction of competing heterologous operators, may prove sufficient to alter lysis versus lysogeny outcomes. Although experiments that investigate and report on such intracellular protein levels will require careful design and execution, they should yield important details on the relationship between *immI*, C2-Cro levels, and ultimately lysis-lysogeny by P22 phage.

Our study investigates the *in vitro* relationship between lysogeny and lysis. Although the frequencies of lysogeny and phage resistance in culture are comparable to those observed in mice, there are likely several mechanisms operating in concert, some of which are possibly altered by deletions in P22 phage, e.g., rate of lysogeny, fitness of lysogens, and comparative rates of bacterial and phage propagation. A quantitative characterization of how conditions in the mammalian gut can alter the dynamics of interaction between phage and bacteria would provide exciting insights on the balance of lysogeny and phage resistance.

## STAR★Methods

### Key resources table

**Table:** 

REAGENT or RESOURCE	SOURCE	IDENTIFIER
**Bacterial and virus strains**		

*Salmonella enterica* subsp. *enterica* serovar Typhimurium 14028s WT (STm)	ATCC	CDC 6516-60
S.Typhimurium K42T	This study	STm, strep^R^
S.Typhimurium Δ*rfbP::KmR*	Michael McClelland	STm resistant to P22
S.Typhimurium Δ*rfbP::CmR*	Michael McClelland	STm resistant to P22
S.Typhimurium P22 WT lysogen	This study	STm P22
S.Typhimurium P22Δ*gtrC-gtrA::kmR* lysogen	This study	STm P22ΔA
S.Typhimurium P22Δ*sieA::kmR* lysogen	This study	STm P22ΔB
S.Typhimurium P22Δ*ninA-ninH::kmR* lysogen	This study	STm P22ΔC
S.Typhimurium P22Δ*orf25-orf80*::*kmR* lysogen	This study	STm P22ΔD
S.Typhimurium P22Δ*mnt-ant::kmR* lysogen	This study	STm P22ΔE
S.Typhimurium P22Δ*xis::GenR* lysogen	This study	STm P22Δxis
S.Typhimurium P22Δ*xis*Δ*orf25-orf80::kmR* lysogen	This study	STm P22ΔxisΔD
S.Typhimurium P22Δ*xis*Δ*mnt-ant::kmR* lysogen	This study	STm P22ΔxisΔE

**Chemicals, peptides, and recombinant proteins**		

Kanamycin	VWR	CAT# 75856-686
Chloramphenicol	Fisher BioReagents	CAT# BP904-100
Gentamicin	Bio Basic	CAT# GB0217-25
Streptomycin	Goldbio	CAT# S-150-100
Mitomycin C	Cayman Chemical Company	CAT# 11435

**Experimental models: Organisms/strains**		

C57BL/6J female mice	The Jackson Laboratory	Strain #:000664

**Oligonucleotides**		

See supplemental materials	This manuscript	Table S1

**Recombinant DNA**		

pKD46	Datsenko & Wanner^19^	N/A
pCP20	Datsenko & Wanner^19^	N/A

**Software and algorithms**		

Prism 9	GraphPad software	https://www.graphpad.com/

### Resource availability

#### Lead contact

Further information and requests for resources and reagents should be directed to and will be fulfilled by the lead contact, Bryan B. Hsu (bhsu@vt.edu).

#### Materials availability

All mutant strains generated in this study can be requested from the lead contact.

#### Data and code availability

• All data reported in this paper will be shared by the lead contact upon request.
• This paper does not report original code.
• Any additional information required to reanalyze the data reported in this paper is available from the lead contact upon request.

### Experimental model and subject details

#### Animals

All animal husbandry and procedures were performed in accordance with institutional guidelines as approved by the Virginia Tech Institutional Animal Care and Use Committee under protocol #20–097. All experiments were performed using 6–7 weeks old C57BL/6J female mice purchased from Jackson laboratory and maintained on standard mouse chow and on a 12 h/12 h light dark cycle in groups of ≤5 mice per cage.

### Method details

#### Bacterial strains and growth conditions

Bacterial strains used in this study are listed in key resources table. *Salmonella enterica* serovar Typhimurium 14028s strains were grown on Luria-Bertani (LB) or MacConkey agar, in LB broth or 0.1X LB broth with shaking (220 r.p.m.) at 30°C, 37°C, or 42°C. Phages were suspended in phage buffer (50 mM tris, 100 mM sodium chloride, 8 mM magnesium sulfate, and 0.01% gelatin, pH 7.4.) unless otherwise noted. For antibiotic selection, ampicillin (100 μg/mL), kanamycin (50 μg/mL), chloramphenicol (25 μg/mL), gentamicin (10 μg/mL), or streptomycin (100 μg/mL) were added when appropriate.

#### Generation of P22 lysogens deletions strains

Deletions of P22 phage genes were performed as previously described.^19^ Briefly, FRT-flanked resistance markers kanamycin (KmR) and gentamicin (GenR) were amplified by PCR from pKD13.The primers used for amplification, detailed in Table S1, include also regions of homology to various regions of P22 phage. Cultures of P22 lysogens containing pKD46 were grown at 30°C in LB supplemented with 10 mM L-arabinose to induce the expression of the λ-red system. Upon reaching an OD_600_ 0.4–0.6, cells were pelleted and rendered electrocompetent by multiple washes with ice-cold 10% glycerol. PCR amplicons were then transformed into the induced electrocompetent cells. Transformants were then selected by plating onto LB kanamycin or LB gentamicin. Deletions were confirmed by Sanger sequencing. When needed the antibiotic resistance genes were eliminated by transforming the strains with pCP20 vector. Induction of antibiotics markers loss was performed by incubating the cultures at 42°C for a minimum of 4 h. The loss of antibiotic resistance genes was also verified by Sanger sequencing. Recombinant P22 prophages were purified from lysogens through plaque assay and reisolation in fresh STm hosts.

#### Lysogeny and phage-resistance *in vitro* and *in vivo* assay

##### *In vitro* assay of bacterial lysogens and resistance

Overnight cultures of STm were back diluted 1:100 in fresh LB broth and incubated at 37°C to a final cell density of 10^8^ CFU/mL. Cultures were centrifuged and cell pellets were washed three times before resuspension in an equal volume of either pre-warmed LB broth or 0.1X LB broth. Cultures were then infected with either wild type P22, P22ΔA, P22ΔB, P22ΔC, P22ΔCD, P22ΔD, P22ΔE, or P22-H5 phage lysates at an MOI of 1, prior to incubation at 37°C with shaking. Culture aliquots were collected at 2, 6, and 16 h and then analyzed for bacterial and phage concentrations by culture. For each biological replicate at each timepoint, 25–50 colonies were picked and tested for lysogeny by either resistance to kanamycin (for recombinant P22 phages) or the presence of prophage integration into the bacterial chromosome (for wild type P22). The same colonies were tested for resistance to P22 phage resistance by cross-streaking against P22-H5. P22 lysogens were identified as KmR, P22-resistant non lysogens as KmS and P22-H5 resistant, and wild type non-lysogens as KmS and P22-H5 susceptible. Phages were quantified from the same aliquots by sterilization with chloroform, centrifugation to remove the bacterial debris and plaque assay.

##### *In vivo* assay of bacterial lysogens and resistance

For enteric *Salmonella* infection, a streptomycin pretreatment model was followed as described previously.^30^ Food and water were withdrawn 4 h before pretreatment of mice with 20 mg of streptomycin sulfate (Goldbio) by oral gavage followed by the return of food and water. For *Salmonella* colonization, food and water were again withdrawn for 4 h followed by oral gavage with 10^7^ cfu of streptomycin-resistant STm K42T strain. STm K42T harbors a point mutation in the amino acid 42 within the rpsL gene which confers resistance to streptomycin. The streptomycin-resistant STm culture used for oral gavage was obtained from exponential bacterial cultures cultivated in LB broth supplemented with streptomycin and subsequently rinsed with phosphate-buffered saline (PBS). After STm gavage, water was returned for 2 h before the administration of 10^7^ pfu phage solution. Phages were prepared by 10-fold dilution into 0.1 M sodium bicarbonate immediately prior to oral gavage. Food was then returned. Mouse weights and health were monitored daily with feces collected on the fourth day post-infection. Fecal pellets were suspended into phage buffer at 50 mg/mL and then serially diluted into the same buffer and plated onto MacConkey agar supplemented with streptomycin. Non-lysogens, P22-resistant non-lysogens, and P22 lysogens of STm were determined by the same patching method described above, using 25–50 single colonies per sample. Phage concentration was determined from fecal suspensions in phage buffer using the same method as described above.

#### Prophage induction assay

Overnight cultures of wild type P22, P22ΔD, and P22ΔE lysogens were back diluted 1:100 into fresh LB broth and incubated at 37°C until an OD_600_ of 0.25 (∼10^8^ cfu/mL). Cells were then washed thrice before resuspension into an equal volume of LB broth that was supplemented with or without 0.5 μg/mL of MMC. After 16 h of culture at 37°C, bacteria and phage concentrations were quantified by methods described above.

#### One-step frequency of lysogeny

Overnight cultures of STm were diluted 1:100 in fresh LB broth and incubated at 37°C with shaking at 220 r.p.m. until an OD_600_ of 0.25 (∼10^8^ cfu/mL). Cells were then washed and resuspended into pre-warmed LB broth or 0.1X LB broth prior to the addition of P22ΔB, P22ΔD, or P22ΔE phage for an MOI of 0.01. After static incubation for 5 min at 37°C, cells were immediately pelleted at 16,000xg for 2 min and then gently resuspended in pre-warmed LB broth or 0.1× LB broth to remove free phage. Cell culture was then continued for an additional 20 min. A portion of each sample was serially-diluted and plated onto LB plates supplemented with kanamycin to quantify the number of lysogens while another portion was treated with chloroform with the supernatant tested by plaque assay to quantify phage.

#### Growth curves

Overnight cultures of STm were back diluted in either LB broth with or without 0.5 μg/mL of MMC to a final cell density of 10^7^ cfu/mL with OD_600_ of 200 μL measured in a 96-well microtiter plate at 5 min intervals for 16 h.

#### Competition assays

##### Co-culture competition between lysogens and non-lysogens

Overnight STm cultures were back diluted to a final OD_600_ of 0.05 with 50 μL of P22-resistant STm (Δ*rfbP*) mixed with 50 μL of a competitor strain (non-lysogen, P22ΔD, P22ΔE, P22Δ(D,xis), or P22Δ(E,xis) lysogens) and 100 μL of LB with or without MMC for a final concentration of 0.5 μg/mL. After 24 h incubation at 37°C, bacteria were enumerated by selective plating onto LB supplemented with chloramphenicol to quantify P22-resistant STm and LB supplemented with streptomycin (non-lysogen STm) or kanamycin (lysogen STm) to quantify the competitor strain. The fitness index of each strain was calculated using the following equation:^57^

$$W=\frac{\log\;M_{\left(P22-resistant\;STm\right)}\;}{\log\;M_{\left(competitor\right)}\;},\text{where}\;M=\frac{{\left[\frac{CFU}{mL}\right]}_{t24}}{{\left[\frac{CFU}{mL}\right]}_{t0}}$$

##### Co-culture competition between lysogens and non-lysogens under phage pressure

Similar to the above protocol, but with slight modifications, overnight STm cultures were back diluted to a final OD_600_ of 0.05. P22-resistant STm(Δ*rfbP*) was diluted an additional 100-fold and then 50 μL was mixed with 50 μL of non-lysogenic STm, 10 μL of phage, and 90 μL of LB broth. The final MOI of phage to non-lysogenic STm was 1. Bacteria quantification and fitness index calculations were conducted in the same manner as described above.

### Quantification and statistical analysis

Statistical analyses were performed using GraphPad Prism 10 using the statistical methods described in the corresponding figure legends.

## Footnotes

Supplemental information can be found online at https://doi.org/10.1016/j.isci.2024.109595.
