# Supplementary material for: Bacterial resistance to temperate phage is influenced by the frequency of lysogenic establishment
Source: iScience. 2024 Mar 27;27(4):109595. doi: 10.1016/j.isci.2024.109595 (PMC11016777; doi:10.1016/j.isci.2024.109595)
Supplement: Document S1. Figures S1–S3 and Table S1 [file mmc1.pdf]

iScience, Volume *27*

Supplemental information

Bacterial resistance to temperate phage is influenced by the frequency of lysogenic establishment

Hiba Baaziz, Rita Makhlouf, Michael McClelland, and Bryan B. Hsu

Supplemental information

Bacterial resistance to temperate phage is influenced by the frequency of lysogenic establishment

Hiba Baaziz[1], Rita Makhlouf,[1] Michael McClelland,[2] Bryan B. Hsu[1]*

Table S1. Oligonucleotides used in this study, related to Method Details.

Phage deletions	Forward Primer (5'-3')	Reverse Primer (5'-3')	Size of genomic fragment deleted (bp)
P22 ΔgtrC-gtrA (A)	*GTTAAATATTCTATAGCTAATTAAACCTAACAACTAT* GTGTAGGCTGGAGCTGCTTC	*TACACTTCAGACCTTTCCGAATCCGCTGATTTTCATAATG* CTGTCAAACATGAGAATTAA	2715
P22 ΔsieA (B)	*TTTTCATGGTATCCTGCACAAAACTAAGGAGGTTGGTGTG* GTGTAGGCTGGAGCTGCTTC	*TATAAGCCAAGGACGGCATTTATTTTATAGTTTTTATGAA* CTGTCAAACATGAGAATTAA	471
P22 ΔninA-ninH (C)	*TAATCGCAGGCCTTTTTATTTGGGGGAGAGGGAAGACATG* CTGTCAAACATGAGAATTAA	*ACGCTGATGTTTTGCCTGGTTAATGCTGGTTACGGTCATA* GTGTAGGCTGGAGCTGCTTC	2256
P22 Δorf25-orf80 (D)	*CTGATGGAAAGACATCGGTTATTGCAGAGGCCATTTAATG* GTGTAGGCTGGAGCTGCTTC	*ATTGATTACGCGGACCACATCATGCTCCGGTAGTGAACAG* CTGTCAAACATGAGAATTAA	904
P22 Δmnt-ant (E)	*TGAACCAGGAAGAGCCAACGAGACTTACTGCTACTTAATG* GTGTAGGCTGGAGCTGCTTC	*CTACGGTGAATTTTGGGCATAAAAAAACCCAGCCGAAGCT* CTGTCAAACATGAGAATTAA	1837
P22 Δxis	*ATGTCATCACCCGCGCTCACCTGGACAGTATGCAGCGGAGATTGAAGTGCCG* AGCTCGAATTGGGGATCTTG	*GAAACAGCGGAGTAAACATGGAATCACACAGCCTCACACTTGATGAGGCC* GGTACCGAGCTCGAATTAGCTTCA	127

Italicized sequences represent regions of homology to P22 phage.

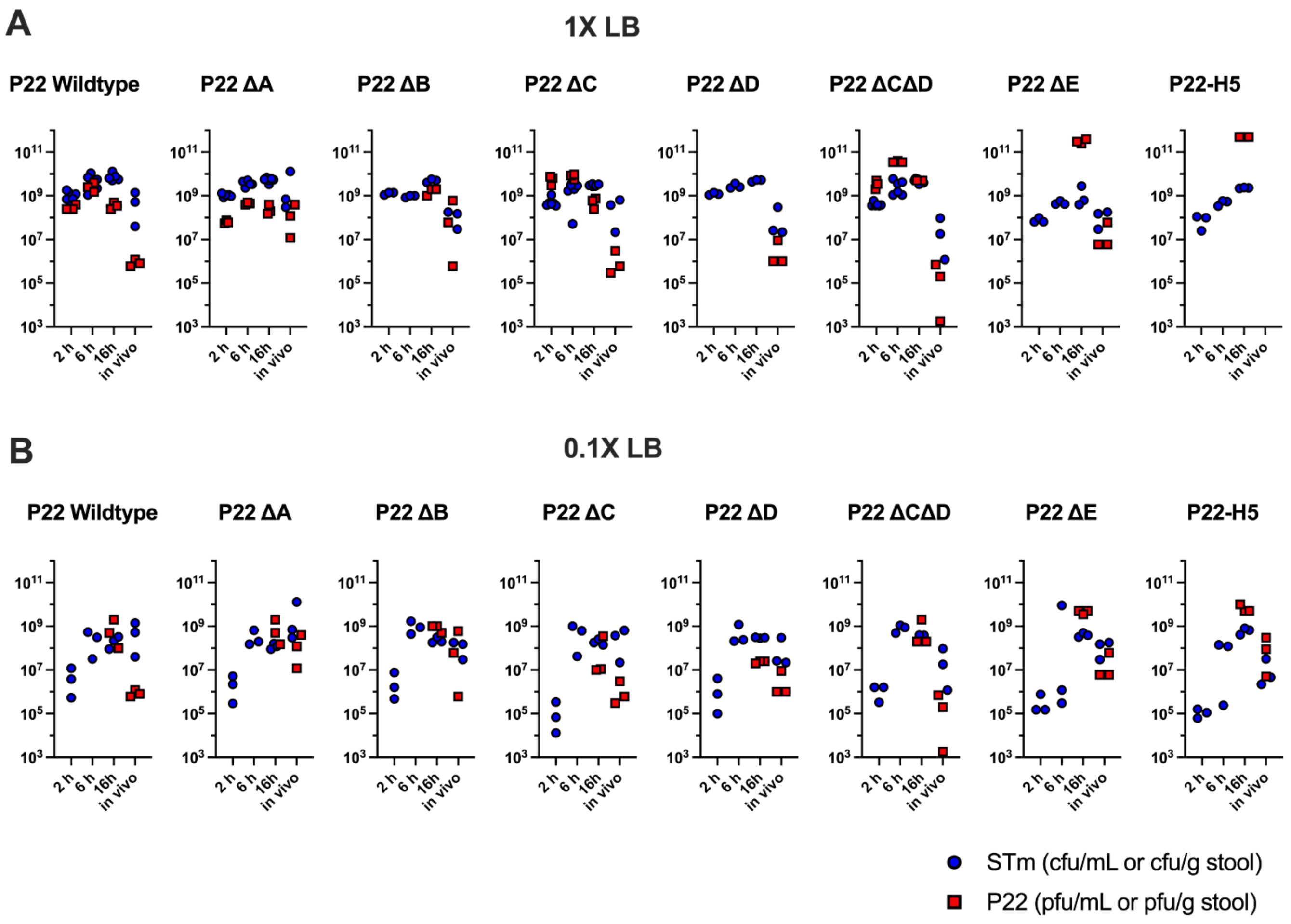

Figure S1. Bacterial and phage concentrations of different cultures, related to Figure 2.

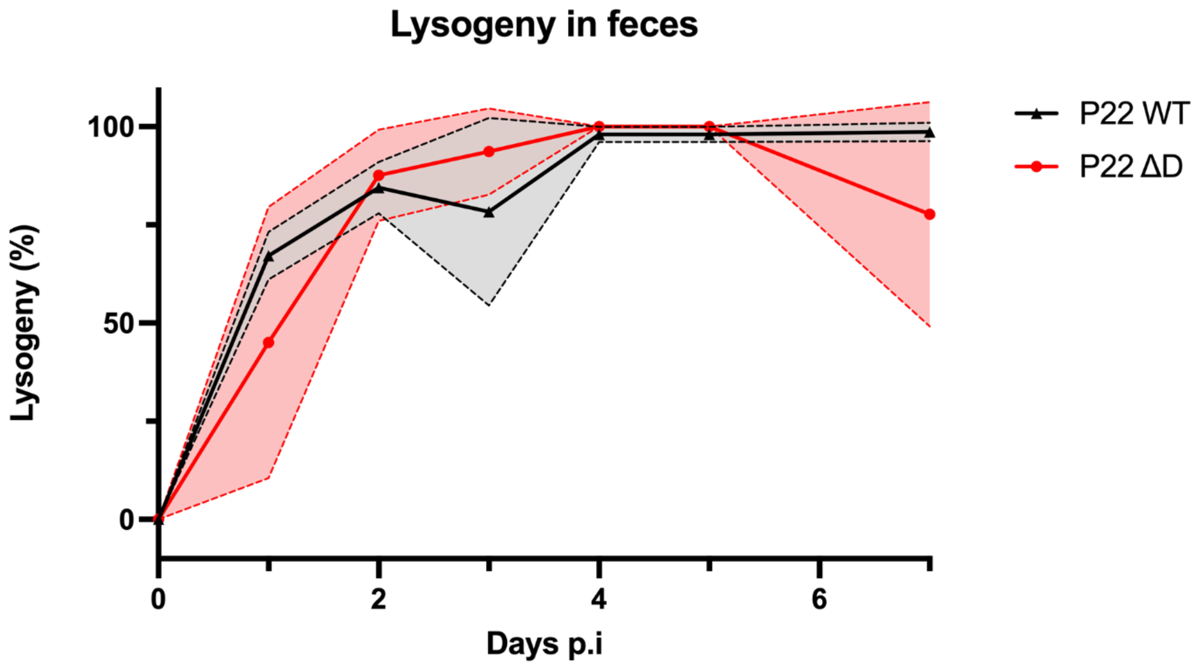

Figure S2. Bacterial lysogeny in murine stool samples over time, related to Figure 2.

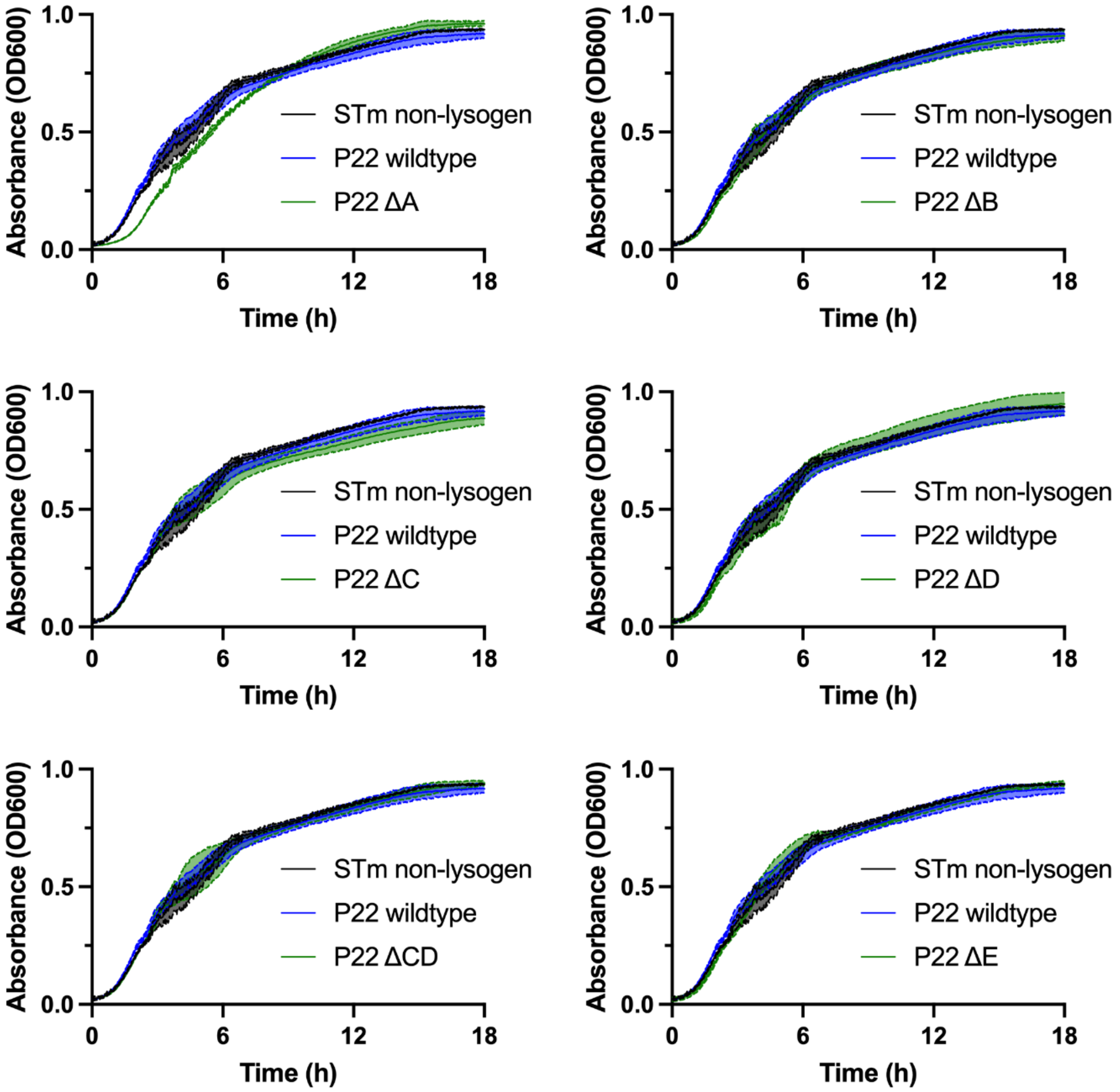

Figure S3. Growth curves of various STm non-lysogen and lysogens *in vitro*, related to Figure 3.
